# Kaposi Sarcoma of the Gastrointestinal Tract in an Adolescent With AIDS

**DOI:** 10.1097/PG9.0000000000000252

**Published:** 2022-10-20

**Authors:** Wesley C. Judy, Elliot Griffith, Pablo Palomo, Jolanda M. Denham, Dorothea Douglas-Lindsay, Lei Shao, Adriana Cadilla, Margaret Donnelly, James P. Franciosi

**Affiliations:** From the * Division of General Pediatrics, Nemours Children’s Hospital, Orlando, FL; † Division of Gastroenterology, Hepatology and Nutrition, Nemours Children’s Hospital, Orlando, FL; ‡ Division of Hematology/Oncology, Nemours Children’s Hospital, Orlando, FL; § Department of Pathology, Nemours Children’s Hospital, Orlando, FL; ∥ Division of Infectious Disease, Nemours Children’s Hospital, Orlando, FL; ¶ University of Central Florida College of Medicine, Orlando, FL.

**Keywords:** Kaposi sarcoma, gastrointestinal tract, endoscopy

## Abstract

Kaposi sarcoma (KS) of the gastrointestinal (GI) tract in a patient with acquired immunodeficiency syndrome (AIDS) has not been reported in an adolescent outside of Africa. We present a 16-year homosexual old male with AIDS, cutaneous KS, pulmonary KS, and gastrointestinal KS (GI-KS) lesions. Eighty percent of patients with GI-KS are asymptomatic, but our patient presented with a month-long history of dysphagia, abdominal pain, and hematochezia. Endoscopy with biopsies revealed multiple KS lesions within the stomach and lower GI tract. This novel case demonstrates the importance of considering early endoscopic screening in immunocompromised adolescents with cutaneous KS to improve morbidity and mortality.

## INTRODUCTION

Kaposi sarcoma (KS) is a rare malignancy caused by the human herpes virus 8 (HHV-8) infection. This angioproliferative tumor most often manifests as multifocal red to purplish cutaneous plaques. Four main types of KS have been identified: classic, endemic, iatrogenic, and AIDS-associated. In the United States, acquisition of KS lesions are most commonly associated with AIDS (1). The incidence of KS has significantly decreased with the introduction of antiretroviral therapy (ART) (2). In the absence of ART, the extracutaneous spread can involve the lymph nodes, liver, lungs, but most often, within the GI tract (1,3).

## CASE REPORT

A 16-year-old homosexual male with a history of asthma who was sexually active presented with multiple cutaneous KS lesions and was found to have an initial human immunodeficiency virus (HIV) RNA viral load of 59,059 copies/mL and a CD4 antigen positive cell count of 58 cells/μL. The patient’s diagnosis of HIV-AIDS with cutaneous KS was made at that time, and the patient was started on daily ART. Baseline endoscopy and colonoscopy were not performed. Despite ART therapy, the patient reported a month history of dysphagia and generalized abdominal pain. In addition, he described several months of watery diarrhea that progressed to hematochezia.

**FIGURE 1.**
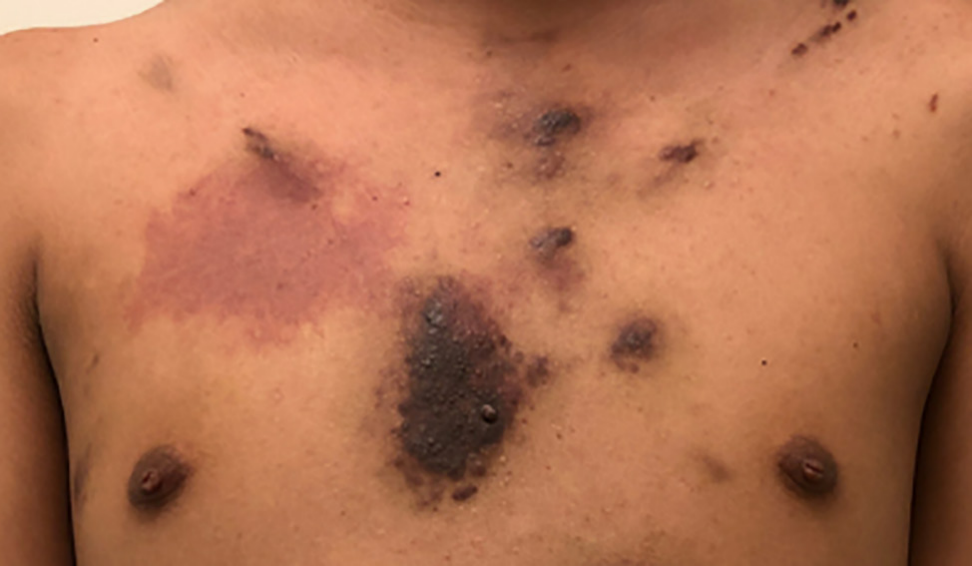
Cutaneous Kaposi sarcoma lesions on chest.

Two months after his initial diagnosis of HIV-AIDS with cutaneous KS, he presented to the emergency department (ED) with respiratory distress, abdominal pain, and hematochezia. In the ED, the physical exam was significant for multiple violaceous plaques on face, chest (Fig. 1), back, arms, groin, legs, and right foot. His lungs had diffuse posterior wheezing bilaterally, and his abdomen was soft, non-tender and non-distended without hepatosplenomegaly. Laboratory studies were significant for hemoglobin of 5.3 g/dL, platelet count of 24 K/µL, HIV RNA viral load < 20 copies/mL, and a CD4 antigen positive cell count of 85 cells/μL. He was started on intravenous (IV) antimicrobial therapy, which was ultimately stopped after blood and urine cultures showed no growth. He was transferred to pediatric intensive care unit (PICU), as he rapidly decompensated into respiratory failure. A chest computed tomography scan (CT) was significant for bilateral pleural effusions and scattered opacities in the lung parenchyma, which was consistent with a superimposed pneumonia.

Over the next 4 days, he stabilized and further evaluation of his gastrointestinal symptoms ensued. The case was reviewed with the NIH. Before proceeding with chemotherapeutic treatments, the NIH recommended a bronchoscopy, and an upper and lower GI endoscopy due to concern for visceral KS manifestations. He continued to have minimal mucus discharge from his rectum, stool hemoccult was positive, and a stool infectious work-up was negative. Abdominal CT was significant for an ill-defined, thick-walled appearance of the cecum and terminal ileum.

**FIGURE 2.**
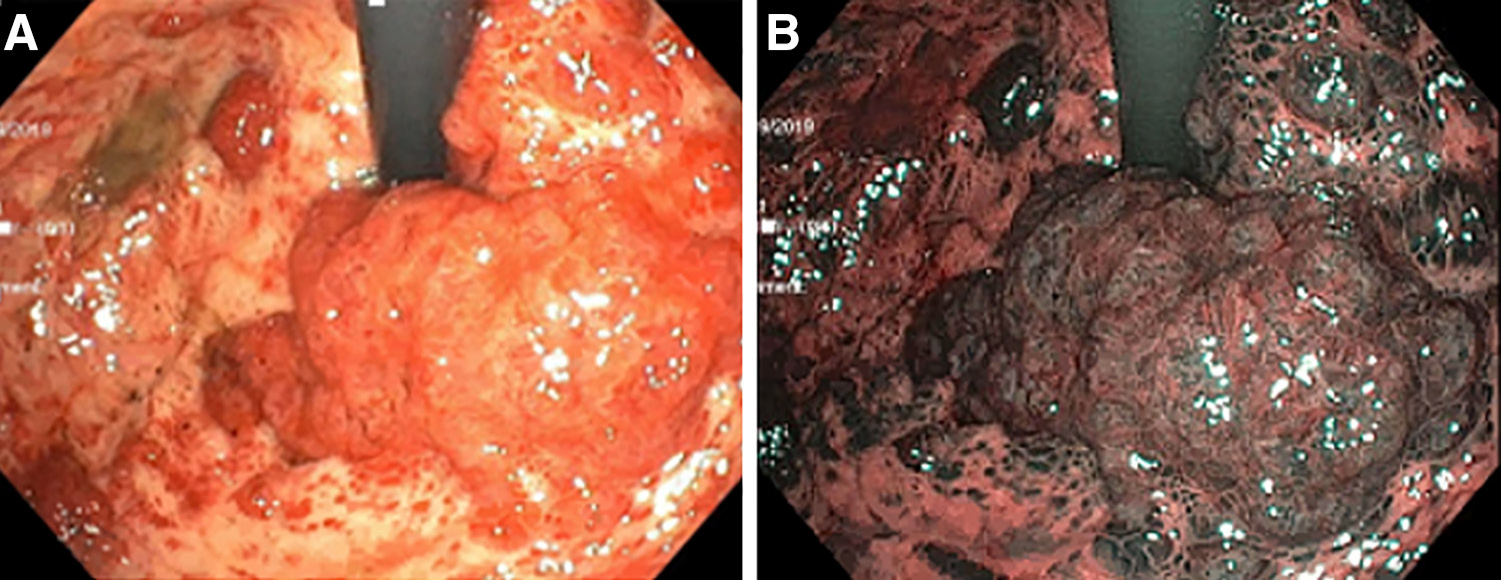
Endoscopic retroflexed (A) view of gastric fundus with KS mass and (B) narrowband image of KS mass. KS, Kaposi sarcoma.

**FIGURE 3.**
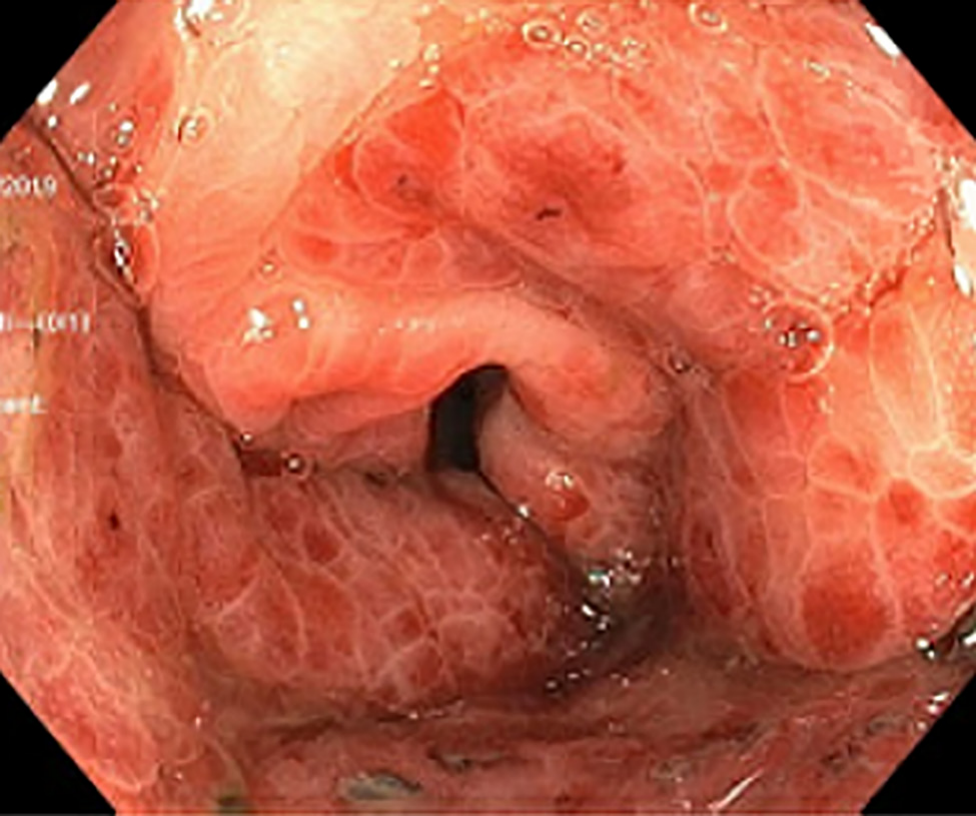
Endoscopic view of circumferential maculopapular KS lesions in patient’s rectum. KS, Kaposi sarcoma.

The upper endoscopy revealed a grossly normal esophagus and duodenum with areas of mild erythema. In contrast, the entire gastric mucosa was covered with superficial maculopapular lesions and multiple polypoid masses—the largest mass was in the gastric fundus (Fig. 2). Due to severely friable gastric mucosa, mild bleeding was observed and easily controlled with argon plasma coagulation after cold forceps biopsies. The lower endoscopy revealed a grossly normal terminal ileum, ascending, transverse, and descending colon. In contrast, the cecum, sigmoid, and rectum (Fig. 3) were significant for a friable mucosa and multiple superficial maculopapular lesions. A minimal amount of blood loss was noted with the lower endoscopy cold forceps biopsies.

**FIGURE 4.**
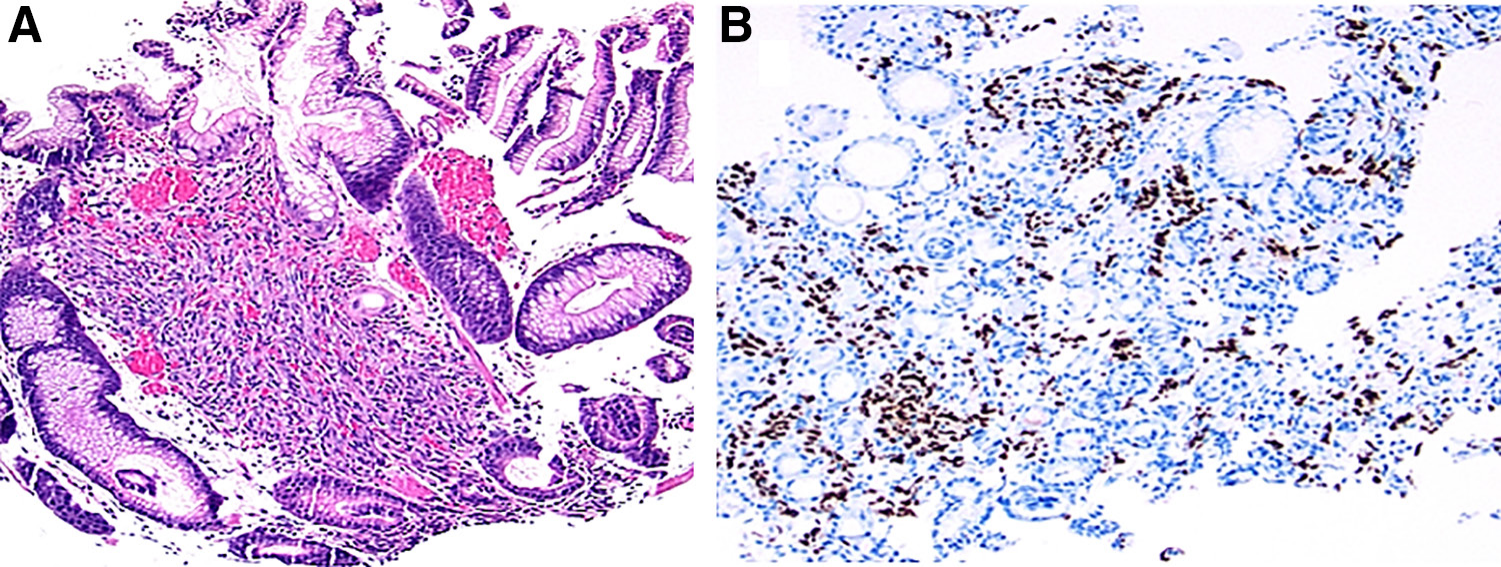
Histology of gastric tissue revealed (A) spindle cell proliferation in the lamina propria (hematoxylin and eosin stain, 100× magnification) and (B) diffusely positive nuclear staining for HHV-8 in the nuclei of the spindle cells (HHV-8 immunohistochemical nuclear stain, 100× magnification).

In the stomach, cecum, sigmoid, and rectum, the histologic examination revealed findings consistent with GI-KS (Fig. 4A)—confirmed with an HHV-8 immunostain (Fig. 4B). Additionally, a bronchoscopy was performed and the quantitative HHV-8 DNA PCR of the bronchoalveolar lavage fluid demonstrated 9300 copies/mL—consistent with pulmonary KS.

The patient continued the daily ART regimen, and started IV liposomal doxorubicin (20 mg/m^2^). His chemotherapy was adjusted in both dosing and agents based on his clinical response. Despite continued medical care and close follow-up, he died 8 months later due to disease progression and opportunistic infections.

## DISCUSSION

A 2014 Cochrane review, “Treatment of KS in children with HIV-1 infection,” examined 4 pediatric cohort studies from individual African countries, as pediatric data are sparse outside Africa. In a disease with a significant risk of morbidity and mortality, the most fortuitous outcomes of KS remission and decreased mortality risk were observed in patients receiving the combination of ART and a chemotherapeutic regimen, in comparison to each treatment alone. However, insufficient pediatric data exists to adequately evaluate the efficacy of these 4 differing chemotherapeutic treatment protocols (4). Due to a lack of evidence, our patient was treated with a combination of ART and the first-line chemotherapeutic agent used in adults with AIDS-KS, liposomal doxorubicin (5).

In AIDS-KS patients, the GI tract is the most common site for extracutaneous KS lesions with an incidence greater than 50% (3,6,10). These lesions manifest in the upper tract at 12% to 24%, the lower tract at 8% to 12%, and both upper and lower tracts at 15% to 20% (9). The predominant GI signs and symptoms are abdominal pain, diarrhea, nausea, and iron-deficiency anemia. Less commonly seen are hemorrhage, perforation, and obstruction (1,6,8,9). However, only 20% of patients with GI-KS exhibit GI signs and symptoms. Most often extracutaneous GI-KS spread is unrecognized, and this insidious manifestation has significant implications on 5-year survival rates (6-9). From 2009 to 2015, those with localized AIDS-KS (including only GI-KS), regional KS spread (i.e., lymph nodes or GI-KS), and distant KS spread (i.e., lungs or liver) have a reported 5-year-survival rate of 82%, 60%, and 38%, respectively. In our patient, although the exact KS origin and spread is not confirmed, we suspect that his cutaneous KS spread to GI-KS and to his lungs. Distant KS spread advanced the TIS (tumor in situ) system staging from T0 to T1 and is associated with a poor-risk outcome (7,11). Patients at highest risk of developing GI-KS were found to be men who have sex with men (MSM), those with low CD4 cell count (<100 cells/μL), high HIV RNA viral load (≥10,000 copies/mL), no history of ART, and presence of cutaneous KS with MSM and low CD4 cell count being the most predictive (10).

Ultimately, treatment regimens rely on the TIS system, as patients with localized disease tend to have better outcomes when compared with more advanced staging. In immunocompromised patients with cutaneous KS, these multiple factors demonstrate the importance of endoscopic screening to enhance early detection and guide treatment in an effort to improve overall morbidity and mortality (7,11).

## ACKNOWLEDGMENTS

All attempts have been exhausted in trying to contact the parents or guardian for the purpose of attaining their consent to publish the Case Report. All identifying information has been removed from this case report to protect patient privacy.
